# Analysis of case management service quality for PCI patients based on the SERVQUAL model: a single-center prospective study

**DOI:** 10.3389/fpubh.2025.1686646

**Published:** 2026-01-06

**Authors:** Songchao Wang, Qin Lu, Li Ding, Weihong Ni, Yeping Zheng

**Affiliations:** 1 Nursing Department, The Second Affiliated Hospital of Jiaxing University, Jiaxing, Zhejiang, China; 2 Department of Cardiology, The Second Affiliated Hospital of Jiaxing University, Jiaxing, Zhejiang, China

**Keywords:** percutaneous coronary intervention, satisfaction, SERVQUAL model, problem focus, importance-performance analysis

## Abstract

**Introduction:**

This study aims to analyze the current status and shortcomings of case management services from the patient’s perspective, investigating percutaneous coronary intervention (PCI) patients’ perceptions and expectations of the quality of case management services. The objective is to provide decision-making references for efficiently improving the quality of case management services.

**Methods:**

Using the Service quality (SERVQUAL) scale as a survey tool, a questionnaire was administered to assess PCI patients’ expectations and actual perceptions of case management service quality, identifying gaps and categorizing findings. Additionally, Importance-Performance Analysis (IPA) was utilized to evaluate PCI patients’ quality assessment of case management services.

**Results:**

A total of 230 PCI patients from August 2024 to July 2025 were surveyed, with 221 valid questionnaires collected. The average expectation score was 3.878, while the average perception score was 3.616, resulting in an average gap of −0.267, indicating an overall moderate service quality. Each dimension’s expectation-perception score gap was negative, revealing that patients’ perceived quality generally fell short of their expectations.

**Conclusion:**

The overall quality evaluation of case management services by PCI patients does not meet their satisfaction level, highlighting the need for continuous quality improvement.

## Highlights

*What is novel?*

- This study uniquely applies the SERVQUAL scale and Importance-Performance Analysis (IPA) to evaluate PCI patients’ perceptions of case management service quality.
- It provides a detailed gap analysis between patients’ expectations and perceptions, highlighting critical areas for improvement.
- The research introduces a patient-centered perspective for assessing case management quality, offering new insights into service evaluation.

*What are the clinical and/or research implications?*

- These findings emphasize the need for targeted interventions to bridge gaps in case management service quality for PCI patients.
- The results serve as a guide for healthcare administrators to prioritize resource allocation and training to enhance patient satisfaction.
- Future research can build on this methodology to assess and improve case management services in other patient populations.

## 1 Introduction

The latest data from China indicate a continuous upward trend in the prevalence of coronary heart disease and the rate of percutaneous coronary intervention (PCI) (1). PCI is a critical revascularization technique for coronary heart disease, though it does not prevent the progression of coronary atherosclerosis; post-PCI, patients remain at risk for in-stent restenosis (2, 3). In alignment with the “Healthy China 2030” planning outline, the Chinese government’s “Medium-to-Long-Term Plan for Chronic Disease Prevention and Control (2017–2025)” (4) emphasizes actively involving society and individuals in chronic disease prevention, fostering healthy behaviors and lifestyles. Case management, a health care system offering assessment, planning, services, coordination, and monitoring for specific diseases, aims to provide coordinated medical and nursing services for specific patient groups (5). Research in developed countries and Taiwan has demonstrated the success of case management as a health care model (6–8). However, a current priority and challenge is helping case managers identify the root causes of quality issues, bridge existing gaps, and improve service quality to enhance patient satisfaction (9).

The SERVQUAL model (service quality), namely the service quality gap model, was first proposed by American marketing experts Parasuraman et al. (10). By analyzing and identifying the gap between the service quality perceived by customers and their expectations, it helps managers identify the root causes of service quality issues, strive to eliminate existing gaps, and thereby improve service quality. It includes five dimensions (tangibility, reliability, responsiveness, empathy, and assurance), which are developed for use in the marketing industry. However, due to its comprehensiveness and practicality, with just a few modifications, any organization can use it. Scholars at home and abroad have attempted to apply the SERVQUAL model to specific scenarios such as hospital outpatient services, inpatient care, and pharmaceutical services, confirming its effectiveness and sensitivity in identifying the shortcomings of medical service quality. In traditional PCI treatment, medical quality assessment mostly focuses on clinical outcomes (such as cure rate and mortality rate) and technical indicators, while relatively neglecting the quality of the service process. A systematic framework is needed to evaluate the “service” itself.

This study, using case management as a perspective and a SERVQUAL model, analyzes the status of case management services for PCI patients. The study proposes optimization solutions for existing standards and management practices, offering strategies and pathways for refined management of PCI patients. It aims to provide practical implementation recommendations and theoretical references for other disease management services.

## 2 Subjects and methods

### 2.1 General information

This study targeted PCI patients admitted from June 2023 to May 2024 as the investigation subjects, and adopted a cross-sectional survey design, aiming to evaluate the perceived quality of service by patients after PCI. The sample size calculation is based on the principle of estimating the accuracy of the confidence interval of the population mean. The significance level (α) is set at 0.05, and the corresponding confidence level is 95%. According to the pre-experiment or previous similar studies (11), the standard deviation (SD) of the expected dimension is approximately 0.9 points, and the error between the expected sample mean and the true population mean (i.e., the half-width of the confidence interval, with an allowable error d) does not exceed 0.125 points. This accuracy is considered to have high practical significance in the field of medical service evaluation. The sample size calculation formula for estimating the population mean is as follows: $n={\left(\frac{AZ_{a/2}\times \sigma }{\mathrm{Ad}}\right)}^{2}$ , where Z_*α*/2_ is the critical value of the standard normal distribution (when α = 0.05, Z = 1.96), *σ* is the expected standard deviation, and d is the allowable error. The calculation results show that to achieve the above accuracy, the theoretical minimum sample size required is 200 cases. This study involves five dimensions of influencing factors. Considering the existence of invalid questionnaires during the investigation process, the sample size was increased by 15%, and thus 230 subjects were included in the investigation.

Inclusion criteria were as follows: (1) patients who agreed to be included in the case management category; (2) ability to use a smartphone; (3) no severe cerebrovascular accidents, mental disorders, or cognitive impairments that could hinder cooperation; (4) informed consent and voluntary participation; and (5) for patients under 18, the survey could be answered by family members, while patients aged 18 to 80 were required to respond independently.

Exclusion criteria were as follows: (1) patients unable to complete the questionnaire due to physical condition; (2) those unwilling to cooperate despite explanations; and (3) those who chose to discontinue participation midway. This study has received ethical approval from the Medical Ethics Committee of Jiaxing Second Hospital (2024–182-01).

### 2.2 Survey method

The SERVQUAL model was initially proposed by American marketing scholars Parasuraman et al. (10). Its core theory is the “service quality gap model,” also known as the “expectation-perception” model, which includes five dimensions: tangibility, reliability, assurance, responsiveness, and empathy. The SERVQUAL model is widely used for evaluating service quality in businesses and has been broadly accepted and adopted by managers and scholars as an effective tool for assessing service quality and enhancing service experience.

This study is based on the theoretical framework of the SERVQUAL model and the specific characteristics of case management services (12). A case management service quality questionnaire based on the SERVQUAL model was utilized, which was translated and adapted into Chinese by Deng et al. (13). The questionnaire includes five dimensions: tangibility (4 items), reliability (5 items), responsiveness (4 items), assurance (4 items), and empathy (5 items), totaling 22 items. The overall Cronbach’s α coefficient of the scale is 0.815, with a test–retest reliability of 0.837, indicating high internal consistency. The scale uses a 5-point Likert scoring method (1 to 5 points representing “very dissatisfied” to “very satisfied”) to evaluate satisfaction and expectations regarding case management services. Service quality is assessed by calculating the difference between patients’ scores on actual service experience and expected service quality. The service quality was calculated as: SQi (service quality) = PSi (service) perception—ESi (service expectations). PSi is the mean score of patient’s perception of the i-th service; ESi is the mean score of patient’s expectation of the i-th service; SQi is the difference between the mean score of patient’s perception of the i-th service and the mean score of expectation.

**Figure 1:**
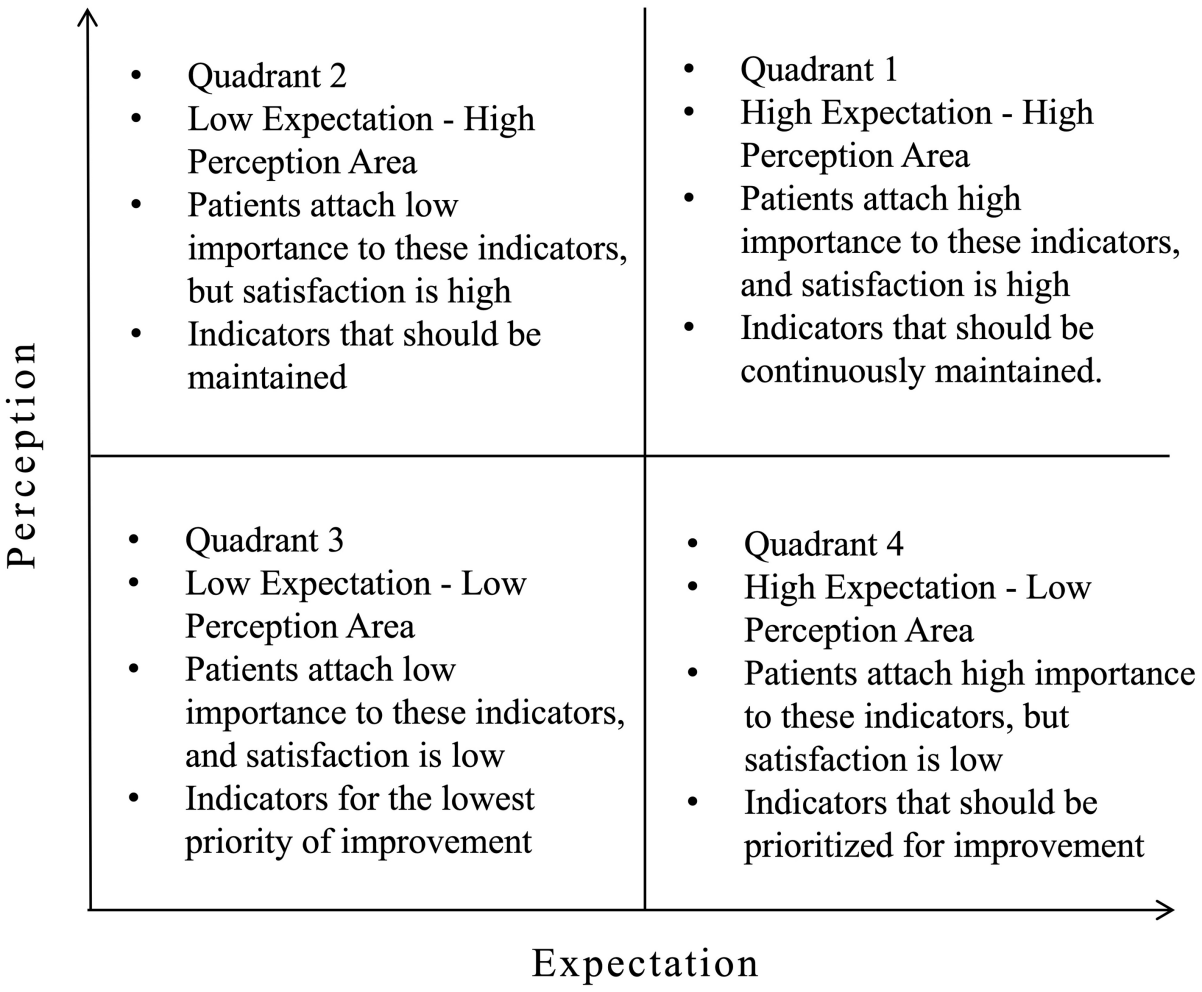
Importance-performance analysis (IPA) quadrantal diagram.

IPA, introduced by Martilla and James (14), is a method for evaluating service quality satisfaction by comparing the importance of customers’ service expectations with the performance of the actual service received. In the IPA model, the average expectation scores for each indicator of case management service quality are plotted on the horizontal axis, while the average perception scores are plotted on the vertical axis. The overall average perception and expectation scores are used as reference lines. All 22 indicators are placed into the four quadrants created by these axes: Quadrant 1 represents high expectation and high perception items, Quadrant 2 represents low expectation and high perception items, Quadrant 3 represents low expectation and low perception items, and Quadrant 4 represents high expectation and low perception items, as shown in Figure 1. This provides a straightforward way to categorize service quality, clearly identifying strengths and weaknesses in resource allocation for quality management and guiding targeted quality improvement.

### 2.3 Quality control

In this study, a paper questionnaire was used for data collection. The two surveyors who participated were uniformly trained to ensure consistency in the survey process. Before the survey, the surveyors explained to participants that it was anonymous and that the results would be used solely for research purposes. Questionnaires were distributed after obtaining the participants’ consent. Surveyors informed the case managers in advance that they should not influence participants when completing the questionnaire. Each questionnaire was completed independently by the participants. If any questions arose, the surveyors could provide clarification but could not offer or imply personal opinions to avoid information bias. The questionnaires were collected on-site by the surveyors, and any questionnaires with missing responses were excluded. Data was entered by both the surveyors and the researchers to ensure accuracy. After all data was entered, it was immediately reviewed again to check for discrepancies and ensure accuracy.

### 2.4 Statistical analysis

Statistical analysis was conducted using SPSS 22.0 software. General information about the participants was described using frequencies and percentages. The quality scores for case management, based on participants’ expectations and actual perceptions, were described using mean values. An IPA analysis chart was constructed to visually highlight the weak areas in case management quality and identify priority areas for improvement.

## 3 Results

### 3.1 Basic information of survey participants

A total of 230 questionnaires were distributed in this study, with 221 valid responses collected, resulting in an effective response rate of 96. Among the participants, there were 119 males (53.85%) and 102 females (46.15%); age distribution included 7 participants aged 31–40 (3.2%), 22 aged 41–50 (10.0%), 73 aged 51–60 (33.0%), and 119 aged 60 or older (53.8%). In terms of educational level, 97 participants (43.9%) had below secondary education, while 124 (56.1%) had at least a secondary education. Monthly household income per capita was as follows: ≤2,000 RMB for 2 participants (0.9%), >2,000–5,000 RMB for 40 (18.1%), >5,000–8,000 RMB for 122 (55.2%), >8,000–10,000 RMB for 54 (24.4%), and >10,000 RMB for 3 (1.4%). Medical insurance coverage included 68 with urban resident insurance (30.8%) and 153 with urban employee insurance (69.2%). There were 98 patients (44.3%) undergoing emergency PCI and 123 patients (55.7%) undergoing elective PCI.

### 3.2 SERVQUAL model analysis results

**Figure 2:**
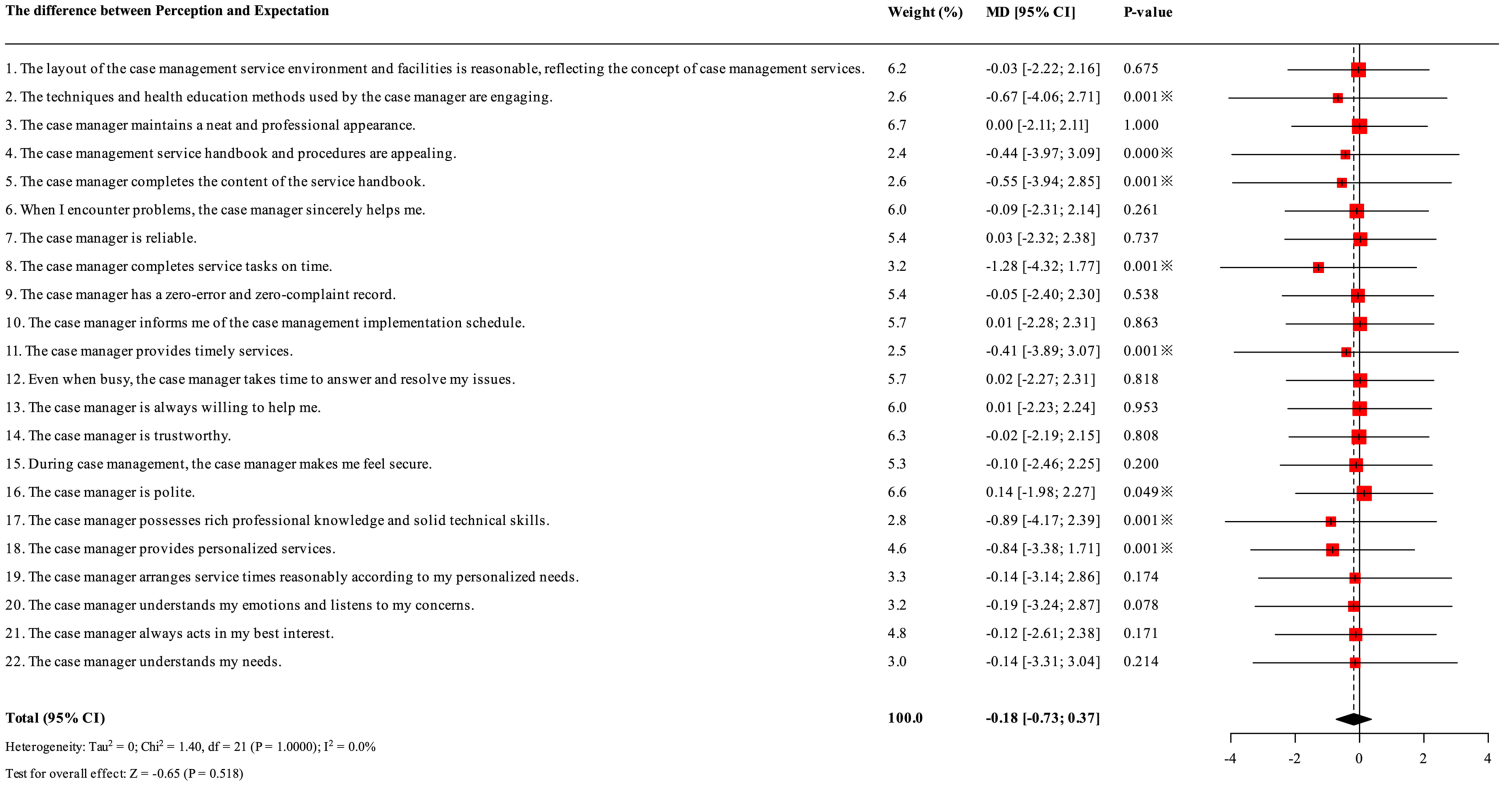
Forest plots of quality rating gaps of case management services (the difference between perception and expectation). This forest plot presents the results of a meta-analysis synthesizing evaluations across 22 specific aspects of case management services. The test for the overall effect showed that the overall effect of case management services was not statistically significant (Z = −0.65, *p* = 0.518). However, items 2, 4, 5, 8, 11, 17, and 18 are statistically significant.

PCI patients had high expectations for case management services, with the mean scores for each dimension ranked as follows: reliability, empathy, tangibility, assurance, and responsiveness (Table 1; Figure 2). The overall mean expectation score for case management was 3.878, while the mean perceived score was 3.616, resulting in an average gap of −0.267. This gap suggests that the effectiveness of case management services has not yet met patient expectations and indicates a need for further improvement and refinement. Among the dimensions, the largest expectation-perception gap was observed in reliability, highlighting it as an area requiring significant attention, followed by empathy. Subgroup analysis revealed that emergency PCI patients scored significantly lower across all dimensions compared to elective PCI patients. Furthermore, older patients (≥60 years) reported significantly poorer service experiences, particularly in Responsiveness and Empathy, highlighting the need for service optimization tailored to specific patient subgroups (Table 2).

**Table 1:** Patient ratings of the quality of case management services (points).

Dimensions	Items	Expectation (E)	Perception (P)	Gap (P-E)	Average
Tangibility	1. The layout of the case management service environment and facilities is reasonable, reflecting the concept of case management services.	3.97	3.94	0.03	−0.27
	2. The techniques and health education methods used by the case manager are engaging.	3.76	3.09	−0.67	
	3. The case manager maintains a neat and professional appearance.	3.99	3.99	0	
	4. The case management service handbook and procedures are appealing.	3.58	3.14	−0.44	
Reliability	5. The case manager completes the content of the service handbook.	3.59	3.04	−0.55	−0.39
	6. When I encounter problems, the case manager sincerely helps me.	4.04	3.95	−0.09	
	7. The case manager is reliable.	3.99	4.01	0.02	
	8. The case manager completes service tasks on time.	4.39	3.11	−1.28	
	9. The case manager has a zero-error and zero-complaint record.	4.02	3.97	−0.05	
Responsiveness	10. The case manager informs me of the case management implementation schedule.	3.95	3.96	0.01	−0.1
	11. The case manager provides timely services.	3.46	3.05	−0.41	
	12. Even when busy, the case manager takes time to answer and resolve my issues.	3.99	4.01	−0.02	
	13. The case manager is always willing to help me.	4.05	4.06	0.01	
Assurance	14. The case manager is trustworthy.	3.98	3.96	−0.02	−0.22
	15. During case management, the case manager makes me feel secure.	4.06	3.95	−0.11	
	16. The case manager is polite.	3.86	4.00	0.14	
	17. The case manager possesses rich professional knowledge and solid technical skills.	4.06	3.17	−0.89	
Empathy	18. The case manager provides personalized services.	4.00	3.17	−0.83	−0.31
	19. The case manager arranges service times reasonably according to my personalized needs.	3.56	3.42	−0.14	
	21. The case manager understands my emotions and listens to my concerns.	3.41	3.22	−0.26	
	22. The case manager always acts in my best interest.	4.05	3.93	−0.19	
	23. The case manager understands my needs.	3.55	3.41	−0.14	
Average		3.878	3.616	−0.267	

**Table 2:** Basic Information of the survey subjects and subgroup analysis of perceived service quality in each dimension of SERVQUAL (*n* = 221).

Characteristic	Category	Number of cases (*n*)	Percentage (%)	Reliability (point)	Prob	Empathy (point)	Prob	Tangibility (point)	Prob	Assurance(point)	Prob	Responsiveness (point)	Prob
Gender	Man	119	53.85%	4.12 ± 0.68	0.142	3.95 ± 0.72	0.016*	4.30 ± 0.61	0.351	4.25 ± 0.65	0.463	4.02 ± 0.74	0.165
	Woman	102	46.15%	4.25 ± 0.59		4.18 ± 0.65		4.22 ± 0.70		4.31 ± 0.58		4.15 ± 0.69	
Age group	Aged 31 to 40	7	3.2%	4.45 ± 0.52	0.023*	4.30 ± 0.48	0.008*	4.52 ± 0.43	0.051	4.48 ± 0.39	0.067	4.40 ± 0.45	0.018*
	Aged 41 to 50	22	10.0%	4.32 ± 0.55		4.20 ± 0.60		4.38 ± 0.55		4.40 ± 0.52		4.28 ± 0.58	
	Aged 51 to 60	73	33.0%	4.20 ± 0.62		4.08 ± 0.68		4.28 ± 0.64		4.28 ± 0.62		4.10 ± 0.70	
	≥60 years old	119	53.8%	4.08 ± 0.70		3.92 ± 0.75		4.18 ± 0.72		4.18 ± 0.68		3.95 ± 0.78	
PCI type	Emergency PCI	98	44.3%	4.05 ± 0.73	0.007*	3.88 ± 0.78	<0.001*	4.15 ± 0.75	0.031*	4.15 ± 0.72	0.009*	3.92 ± 0.81	0.003*
	Elective PCI	123	55.7%	4.28 ± 0.58		4.20 ± 0.62		4.35 ± 0.59		4.38 ± 0.55		4.20 ± 0.65	
Totality		221	100.0%	4.18 ± 0.65		4.05 ± 0.70		4.26 ± 0.66		4.28 ± 0.62		4.08 ± 0.72	

*Indicates that under this feature, the score differences among different subgroups are statistically significant (*p* < 0.05).

### 3.3 IPA analysis results

**Figure 3:**
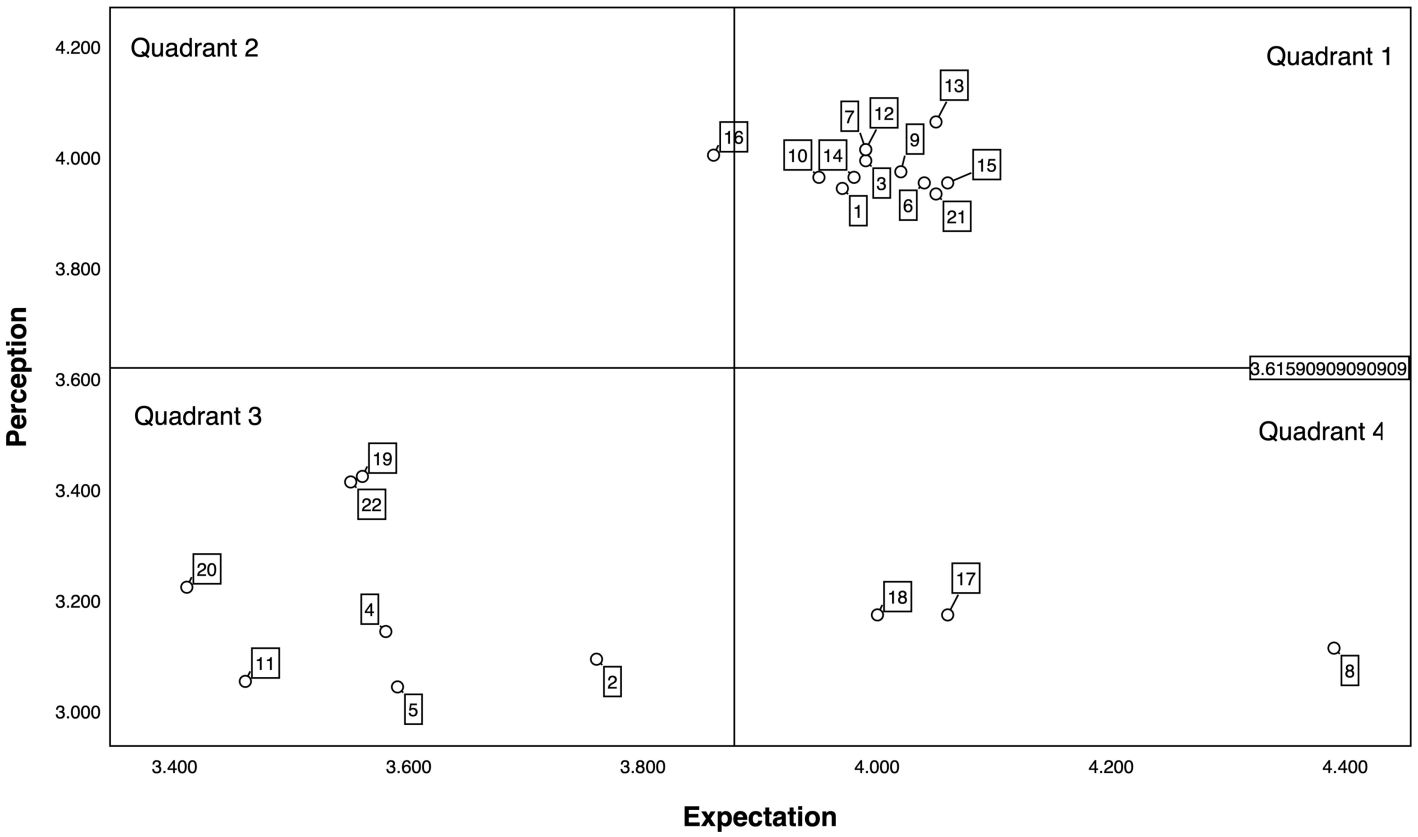
Importance-performance analysis (IPA) results of patients after percutaneous coronary intervention (PCI). (1) The layout of the case management service environment and facilities is reasonable, reflecting the concept of case management services. (2) The techniques and health education methods used by the case manager are engaging. (3) The case manager maintains a neat and professional appearance. (4) The case management service handbook and procedures are appealing. (5) The case manager completes the content of the service handbook. (6) When I encounter problems, the case manager sincerely helps me. (7) The case manager is reliable. (8) The case manager completes service tasks on time. (9) The case manager has a zero-error and zero-complaint record. (10) The case manager informs me of the case management implementation schedule. (11) The case manager provides timely services. (12) Even when busy, the case manager takes time to answer and resolve my issues. (13) The case manager is always willing to help me. (14) The case manager is trustworthy. (15) During case management, the case manager makes me feel secure. (16) The case manager is polite. (17) The case manager possesses rich professional knowledge and solid technical skills. (18) The case manager provides personalized services. (19) The case manager arranges service times reasonably according to my personalized needs. (20) The case manager understands my emotions and listens to my concerns. (21) The case manager always acts in my best interest. (22) The case manager understands my needs.

As shown in Figure 3, items 2, 4, 5, 11, 19, 20, and 22 are located in the third quadrant, while items 8, 17, and 18 fall into the fourth quadrant, indicating areas that require improvement and refinement in service quality measurement.

## 4 Discussion

### 4.1 Quadrant One: sustain and maintain

Quadrant One represents areas of strength, highlighting items where case management service performance is relatively high compared to the origin average of expectation-perception. Key items include “case management service environment and facility layout is reasonable and reflects the concept of case management,” “case managers maintain a neat appearance,” “case managers sincerely help me when I encounter issues,” “case managers are reliable,” and “case managers have a zero-error, zero-complaint record.” These items suggest that case managers place great importance on patient interactions and uphold strong ethical standards. Quadrant One includes five dimensions, with reliability and responsiveness containing the most items (three each), indicating these dimensions perform best. This aligns with Aghamolaei et al.’s (15) findings, which identified reliability as the most critical aspect in assessing hospital services. Patients feel significant trust in case managers due to their high medical and ethical standards. The assurance dimension includes two items: “case managers are trustworthy” and “case managers provide me with a sense of security,” showing high patient satisfaction with these aspects. The 11 items in Quadrant One should be maintained and strengthened in future adjustments to continuously enhance case management quality.

### 4.2 Quadrant Two: moderate adjustment

Items in Quadrant Two indicate areas where patient expectations are low, but post-care satisfaction is high, resulting in relatively high service satisfaction. This quadrant includes one item, “case managers are polite,” which enhances the patient’s experience. Although patients do not prioritize politeness, they still value it, as it fosters harmonious interpersonal relationships. During patient-caregiver interactions, patients tend to be psychologically vulnerable and sensitive to language, emphasizing the importance of politeness to promote communication and understanding, thereby establishing a balanced, cooperative caregiver-patient relationship.

### 4.3 Quadrant Three: continuous improvement

Quadrant Three represents items where both expectation and perception scores are below the origin average, indicating less patient attention in these areas but substantial room for improvement. Items in this quadrant primarily involve tangibility, such as “case managers use engaging techniques and health education formats,” “the case management service handbook and procedures are attractive,” and “case managers complete the handbook content.” PCI patients generally focus on the severity of their condition, so they are less concerned with these aspects and consequently have lower expectations. However, when “case managers provide timely services,” patients are highly dependent on medical staff. As patients’ awareness of preventive care increases, they need professional nursing to promote recovery, resulting in a greater demand for ongoing care, similar to findings by Lanjun et al. (16). The empathy dimension also includes three items that require enhanced awareness among medical staff to reflect a patient-centered approach, thereby providing the best service experience. This need for improvement in empathy aligns with Andaleeb’s (17) findings, which suggest that empathy is a critical area for improvement in case management to enhance the patient experience.

### 4.4 Quadrant Four: priority improvement

Items in Quadrant Four are high in patient expectations but low in perception, indicating that patients consider these aspects important and believe they should be well-executed, yet their actual experience falls short. Possible reasons include lack of interaction or perceived lack of service after interaction. One item each from reliability, assurance, and empathy falls into this quadrant, with the largest expectation-perception gaps observed in “case managers complete services on time,” “case managers possess rich professional knowledge and strong technical skills,” and “case managers provide personalized service.” Patients have high expectations for receiving timely services, but gaps in service content reduce their perception scores. Case managers’ knowledge and skills are fundamental to implementing effective case management, requiring not only extensive clinical experience but also strong management, communication, and literature review abilities (18). In Taiwan’s clinical practice, each oncology case manager is responsible for managing over 400 cases, likely far exceeding the workload of case managers in other countries (19). This highlights the need for information system support to assist case managers in smart statistics and personalized management, an area requiring critical improvement for case managers.

## 5 Recommendations

### 5.1 Sustaining the advantages of case managers’ medical ethics and conduct

To ensure that case managers’ behavior adheres to high standards, hospital party-building initiatives should be integrated with the development of medical ethics and conduct. This will support a well-defined behavior management system for healthcare professionals, allowing case managers to follow established ethical guidelines. A robust, long-term mechanism for supervision and evaluation should be maintained. Meanwhile, a people-centered development philosophy should be upheld, respecting and protecting patients’ social, psychological, and emotional needs, and safeguarding their basic rights from infringement.

### 5.2 Enhancing case management systems and standardized management

First, case management should address three main stages and five key phases: the three stages—case initiation, case management, and case closure—and the five phases—assessment, planning, implementation, evaluation, and feedback. Effective service and management strategies should be tailored according to the specific diseases and patient groups. For example, a “traffic light” model can be adopted for PCI patients’ dietary prescriptions, with food images in educational materials marked by color (red for high-calorie foods, yellow for moderate-calorie foods, and green for low-calorie foods) and energy requirements based on patients’ activity levels (20). Through color-coded nutritional labels, patients can easily make appropriate dietary choices.

In addition, case management services and management processes can be adapted as needed. For instance, monitoring timely service completion and maintaining standardized management can maximize case managers’ strengths. Internet platforms like WeChat can be used to streamline services: patients and their families can scan a QR code under nurse guidance to enroll and create an initial electronic medical record (EMR). Case managers can log in to the medical interface to complete the EMR details.

Through this platform, case managers can conduct targeted follow-ups after discharge based on each patient’s specific conditions, tracking physical health, diet, sleep, mental state, medication, daily activities, and psychological well-being. They can inquire about post-discharge exercise routines, nutrition, and sources of psychological distress, offering tailored support and health education accordingly. Regular updates on cardiac rehabilitation resources can also be sent. Patients and their families can use the platform to consult with the team and receive timely responses, establishing an internet-based, standardized management system to reduce challenges related to accessibility and limited patient engagement, which can impede service completion.

Given the trend of cardiovascular diseases affecting younger patients, Nabutovsky et al. (21) compared the impact of mobile health (mHealth) applications on younger and older patients, finding that younger patients showed greater interest in lifestyle recommendations and higher acceptance of remote cardiac rehabilitation. By incorporating AI and big data, a standardized monitoring database can be developed, allowing high-quality healthcare resources to reach more remote areas. Items 2, 4, and 5 fall into the third quadrant of the IPA analysis chart, indicating that traditional paper-based health education materials do not fully meet patients’ needs, and there is room for improvement and enhancement in the standardized services for PCI patients.

### 5.3 Actively exploring and enhancing case management service content

The study results indicate that participants place high importance on “the case manager’s ability to understand my emotions and listen to my concerns,” highlighting the need for timely adjustments and improvements in this area. Currently, preoperative PCI education is often abstract, monotonous, and lecture-based, which can be difficult for patients and families with high psychological burdens or limited comprehension skills to fully understand. Despite good interventional procedures, a lack of disease knowledge may lead to somatic symptomatology. To improve education, we could adopt methods such as image simulation, object simulation, or video simulation, making the learning process more engaging, accessible, memorable, and less rigid. This approach is especially beneficial for older patients or those with memory challenges.

After PCI, patients may experience ongoing concerns about the stent, the risk of in-stent restenosis, or potential issues with other vessels. Symptoms like chest discomfort may persist, and lack of family, economic, or social support can adversely affect their mental state, potentially leading to or exacerbating depression. Beyond alleviating negative emotional experiences, case managers should work to improve patients’ health literacy regarding exercise. Keessen et al. (22) found that many patients experience a fear of exercise following acute coronary syndrome, while the American College of Sports Medicine recommends that, in the absence of limiting health conditions, older patients with coronary heart disease should combine a healthy diet with at least 150 min of moderate physical activity per week to help optimize energy balance and slow disease progression (23). Subgroup analysis revealed significant disparities in service quality perceptions. Systematic differences in patient experience were observed across PCI types, with advancing age showing a negative correlation with service ratings. Additionally, gender-based differences emerged specifically in the “Empathy” dimension. However, a survey of PCI patients over 60 revealed that over 70 avoid exercise due to concerns about adverse cardiovascular events (24). Therefore, case managers should emphasize the benefits of physical activity, encourage patients to participate, and teach them self-assessment techniques to select suitable forms of exercise based on their condition, such as table tennis or tai chi. Personalized exercise approaches not only support active rehabilitation but also help relieve psychological stress.

### 5.4 Strengthening the continuity of case management services

The service attitude and management level of case managers should be based on PCI patients’ needs and experiences, with prompt responses to changes in case management. Designing service workflows carefully and establishing standardized training systems will provide structured training for case managers, improving both service and management skills. A record of individual training progress should be maintained, allowing case managers to identify areas for improvement and enabling management to assess training effectiveness and competency levels. By minimizing discrepancies due to varying service standards, case management services can achieve seamless and unified operations, providing patients with standardized and professional case management experiences. This will enhance PCI patients’ satisfaction and recognition of case management services and improve overall service quality.

### 5.5 Optimizing feedback channels for PCI patients

Effective communication is not measured by how well both parties explain the matter, but by how well they understand each other. The management of services for PCI patients can be communicated through various methods, such as ward bulletin boards, posters, and handbooks, to present information to patients. It is also crucial to accurately gather and understand patients’ suggestions and opinions on case management services. Case management providers can effectively utilize convenient channels, such as social networks, to gather patients’ needs and feedback promptly. By fully understanding first-hand feedback on case management services, multiple feedback channels can be established, enabling targeted measures and services tailored to patients’ actual situations. This continual improvement of service quality enhances patient engagement and satisfaction, fulfilling the principle of “starting with patient needs and ending with patient satisfaction,” which effectively protects patients’ interests and supports the sustainable development of case management.

## 6 Conclusion

In summary, this study constructed a case management service quality evaluation index system using the SERVQUAL-IPA model, tailored to the attributes of case management services. The results reveal certain disparities in the overall evaluation of case management service quality among PCI patients, with substantial differences in scores for reliability and empathy, while the dimensions of tangibility, assurance, and responsiveness showed smaller gaps. These findings provide a reference for identifying issues within different dimensions of case management services, aiding the high-quality development of services for PCI patients.

### 6.1 Limitations of the study

The SERVQUAL-IPA model used in this study evaluates service quality through the gap between patients’ perception and expectation. Although it is widely applicable, it is difficult to fully reflect the actual clinical effect of case management because it only relies on subjective evaluation. In the future, objective data such as postoperative complication rate, medication compliance, readmission rate and quality of life should be integrated to establish a more scientific evaluation system that integrates subjective and objective information. Because the data comes from a single medical institution, the applicability of the research results in other settings is limited due to its management model, resource allocation and patient population characteristics. There are significant differences in cardiovascular diagnosis and treatment ability, nursing staff allocation and informatization level of hospitals in different regions and grades in China, which makes it difficult to directly popularize this research tool and strategy. However, as the first exploratory study to apply the international universal service quality model to the case management of PCI patients in China, it not only provides a local practical perspective, but also shows that when introducing foreign management frameworks, the Chinese cultural background, doctor-patient communication methods and the characteristics of medical system should be fully considered. In the follow-up, multi-center collaboration should be carried out, based on large sample data, combined with machine learning and potential category analysis, to build a service quality standard with both cultural adaptability and Chinese context fit, and promote the development of a patient-centered high-quality cardiac rehabilitation system.

## Data Availability

The original contributions presented in the study are included in the article, further inquiries can be directed to the corresponding author. Requests to access the datasets should be directed to dingli202508@163.com.
